# Publisher Correction: Collective molecular switching in hybrid superlattices for light-modulated two-dimensional electronics

**DOI:** 10.1038/s41467-018-05541-6

**Published:** 2018-09-06

**Authors:** Marco Gobbi, Sara Bonacchi, Jian X. Lian, Alexandre Vercouter, Simone Bertolazzi, Björn Zyska, Melanie Timpel, Roberta Tatti, Yoann Olivier, Stefan Hecht, Marco V. Nardi, David Beljonne, Emanuele Orgiu, Paolo Samorì

**Affiliations:** 1 University of Strasbourg, CNRS, ISIS UMR 7006, 8 allée Gaspard Monge, F-67000, Strasbourg, France; 2 Laboratory for Chemistry of Novel Materials, Center for Research in Molecular Electronics and Photonics, University of Mons, Place du Parc 20, 7000 Mons, Belgium; 3 Department of Chemistry and IRIS Adlershof, Humboldt-Universität zu Berlin, Brook-Taylor-Str. 2, 12489 Berlin, Germany; 4 Department of Industrial Engineering, University of Trento, Via Sommarive 9, 38123 Trento, Italy; 5 IMEM-CNR, Institute of Materials for Electronics and Magnetism, Trento unit, Via alla Cascata 56/C, Povo, 38123 Trento, Italy; 6 Centro de Fisica de Materiales (CSIC-UPV/EHU), Paseo Manuel de Lardizabal 5, E-20018 Donostia, San Sebastián, Spain; 7 Present Address: Department of Chemical Sciences, University of Padua, Via Francesco Marzolo 1, 35131, Padova, Italy; 8 Institut National de la Recherche Scientifique (INRS), EMT Center, Boulevard Lionel-Boulet, Varennes, QC, J3X 1S2, 1650, Canada

Correction to: *Nature Communications* 10.1038/s41467-018-04932-z; published online 09 July 2018

The original version of this article incorrectly listed an affiliation of Sara Bonacchi as ‘Present address: Institut National de la Recherche Scientifique (INRS), EMT Center, Boulevard Lionel-Boulet, Varennes, QC, J3X 1S2, 1650, Canada’, instead of the correct ‘Present address: Department of Chemical Sciences, University of Padua, Via Francesco Marzolo 1, 35131, Padova, Italy’. And an affiliation of Emanuele Orgiu was incorrectly listed as ‘Present address: Department of Chemical Sciences, University of Padua, Via Francesco Marzolo 1, Padova, 35131, Italy’, instead of the correct ‘Present address: Institut National de la Recherche Scientifique (INRS), EMT Center, Boulevard Lionel-Boulet, Varennes, QC, J3X 1S2, 1650, Canada’. This has been corrected in both the PDF and HTML versions of the article.

