# Supplementary material for: Collective molecular switching in hybrid superlattices for light-modulated two-dimensional electronics
Source: Nat Commun. 2018 Jul 9;9:2661. doi: 10.1038/s41467-018-04932-z (PMC6037738; doi:10.1038/s41467-018-04932-z)
Supplement: Supplementary file 1 — Supplementary Information [file 41467_2018_4932_MOESM1_ESM.pdf]

Supplementary Information

Collective molecular switching in hybrid superlattices for light-modulated two-dimensional electronics

M. Gobbi *et al.*

Table of contents

Supplementary Figure 1. 2
Supplementary Figure 2. 4
Supplementary Figure 3. 5
Supplementary Figure 4. 7
Supplementary Figure 5. 9
Supplementary Figure 6. 11
Supplementary Figure 7. 13
Supplementary Figure 8. 14
Supplementary Figure 9. 15
Supplementary Figure 10. 17
Supplementary Note 1 18
Supplementary Note 2 19

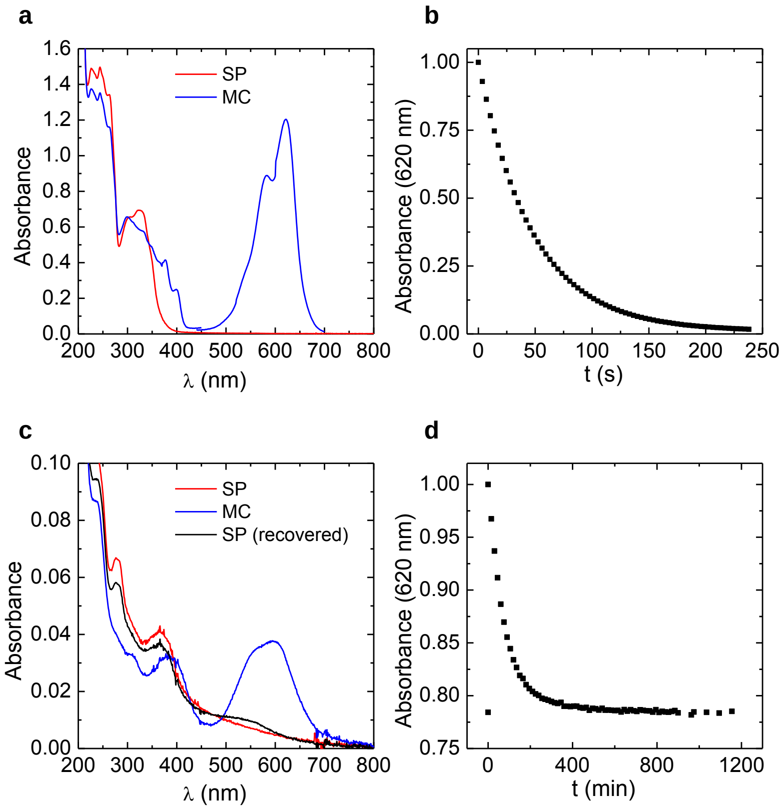

Supplementary Figure 1. Optical characterization of the spiropyran/merocyanine derivative. (**a**) Absorbance spectra measured for the spiropyran (SP, shown in red) / merocyanine (MC, in blue) solution in cyclohexane (0.05 mg mL^{-1}). The MC spectrum was recorded immediately after UV irradiation of the SP solution, and it displays the typical structured band of MC groups centered at 600 nm. In the MC case, the measurement was acquired in two successive intervals (200 – 400 nm, and 400 – 700 nm), and the solution was re-irradiated between two measurements to contrast the quick thermal recovery of the SP isomer. (**b**) Evolution of the solution absorbance (at 620 nm) normalized at the value at t = 0 s after UV irradiation showing the kinetics of the MC→SP relaxation in solution at room temperature. Under these conditions, most MC isomers have relaxed back to the SP form in a few seconds. These optical characteristics are analogous to those reported for SP/MC derivatives without long alkyl chain, indicating that its presence does not change significantly the photochromic response of our derivative. (**c**) Absorbance spectra measured for a SP/MC film, as obtained by spin-coating a 1 mg mL^{-1} solution in cyclohexane on a quartz slide. The spectrum of the SP film was

measured directly after spin-coating, while that of the MC film was recorded after UV irradiation of the same film. As expected, the spectra in the solid state show less structured absorbance bands as compared to those in solution, due to the π-π interaction among molecules. The spectrum of the same film was also measured after 48 h, showing an almost complete recovery. (**d**) Evolution of the film absorbance (at 620 nm) normalized at the value at t = 0 s after UV irradiation at room temperature. As compared with the MC→SP relaxation in solution, the thermal recovery in films is much slower, and a full recovery of the SP state is achieved only after approximately 20 h. The relatively slow MCSP recovery in thin films warrants us with sufficient time to explore the electrical properties of the metastable MC/2DM system (see main text).

For these experiments, UV irradiation was performed with an ultraviolet lamp (UV-6 L/M Herolab) with λ_{irr}= 365 ± 5 nm and a power density 1.7 mW cm^{-2}. The irradiation time for triggering the SP→MC isomerization was 1 min for the solution and 30 min for the spin-coated film.

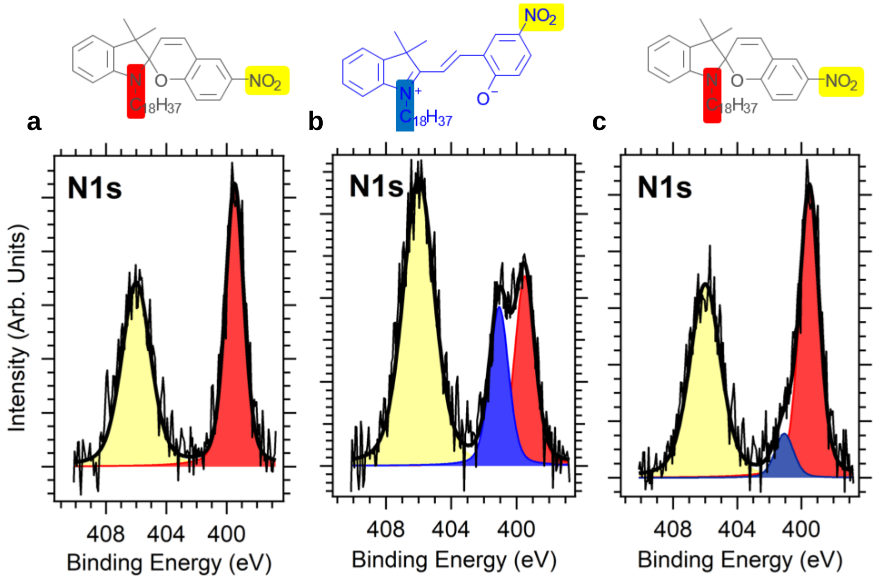

Supplementary Figure 2. Binding energy of N 1s in assemblies on MoS_2. The X-Ray Photoelectron Spectroscopy analysis of the binding energy of the N 1s and O 1s core levels provides unambiguous identification of the isomer on the surface, due to the very different hybridization of the indoline N in SP and MC. (**a-c**) N 1s binding energy measured (**a**) on a film spin-coated on MoS_2 kept in dark, (**b**) on the same film after in situ UV irradiation, (**c**) after subsequent in situ irradiation with green light. Each spectrum is characterized by multiple peaks fitted by different components, corresponding to the different N hybridization. The colors of the fitting components recall those of the N atoms in the chemical structure reported above. The evolution of the spectral characteristics of N is analogous on films spin-coated on HOPG (shown in Fig. 1b-d in the main text) and MoS_2.

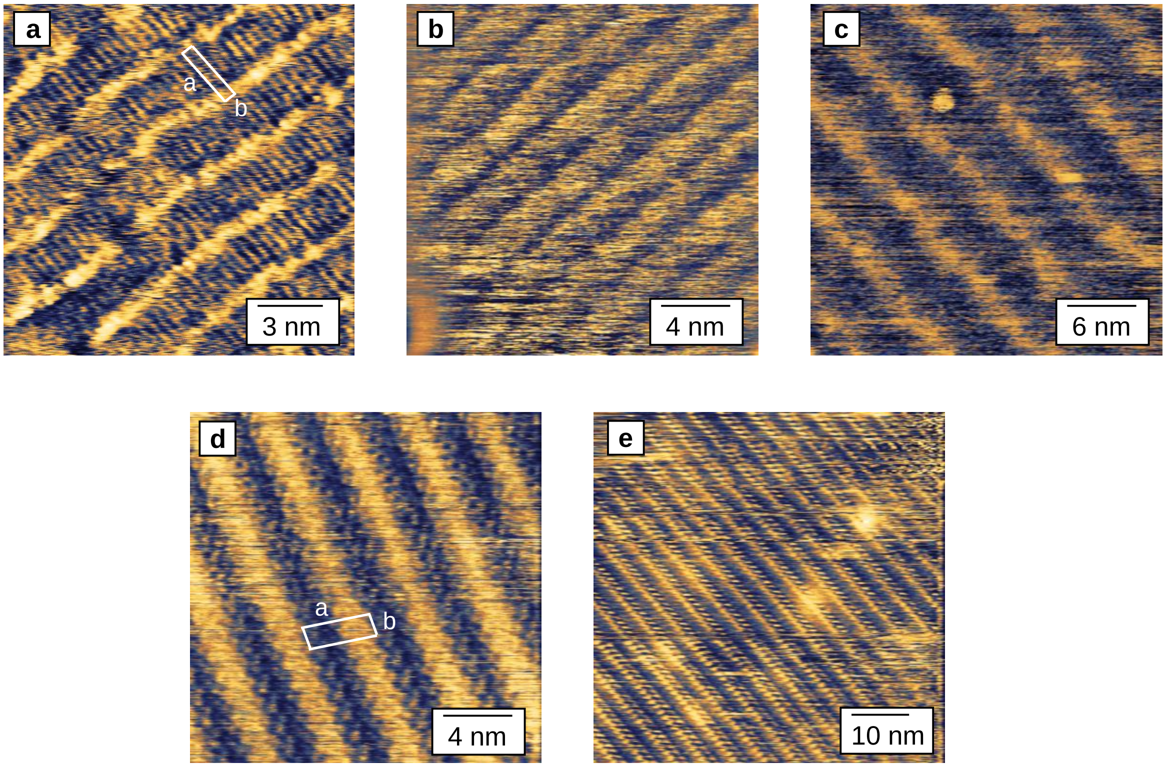

Supplementary Figure 3. Nanoscale assembly of spiropyrans and merocyanine on different substrates. (**a**) Scanning tunnelling microscopy image of a spiropyran (SP) monolayer on HOPG. The assembly shown in this image is different from that presented in Fig. 1e of the main text, being characterized by a unit cell that can be extracted with the following parameters: a = 2.8 ± 0.2 nm, b = 0.45 ± 0.1 nm, α = 82 ± 3°, leading to an area A = 1.25 ± 0.3 nm^2. In particular, the width of the lamellas in this image is half of the one highlighted in the main text. This packing was observed in measurements carried out on four different days, while the assembly shown in the main text was observed on eight different days in different samples prepared in the same way. We ascribe this difference to a head-to-head molecular arrangement, instead of the head-to-tail arrangement shown in Fig. 1. We highlight that even in the assembly shown in (**a**) the head groups are imaged with low resolution, indicating that the SP groups are not orderly aligned, and their dipoles are randomly oriented. (**b-c**) Nanoscale ordering of SP isomer on MoS_2, as measured by STM in air in dry films. The quality of the MoS_2 surface was found to be lower than that of HOPG, leading to a lower overall resolution. Even in this case, two different assemblies were imaged in different samples, one with a lamella width l = 2.4 ± 0.3 nm (**b**) and

the other one with a lamella width $l = 5.9 \pm 0.3$ nm (**c**). (**d-e**) STM images of the merocyanine (MC) assembly on MoS_2, characterized by a unit cell $a = 3.8 \pm 0.3$ nm, $b = 1.2 \pm 0.1$ nm, $\alpha = 82 \pm 3°$, leading to an area $A = 4.7 \pm 0.6$ nm^2, in close agreement to that measured on HOPG. In order to increase the resolution, images (**d**) and (**e**) were measured at the solid/liquid interface, by covering the MoS_2 surface with a 4 μL drop of a 1 mg mL^{-1} SP solution in phenyloctane, UV irradiated immediately before the measurement. The SP assembly could not be imaged on at the solid/liquid interface. The height channel is shown for all images, except for (**b**), for which the lamellar ordering could be better appreciated in the displayed current channel. The images on MoS_2 could not be rescaled to account for the drift in the piezo, as the atomic resolution of the underlying MoS_2 lattice could not be obtained reproducibly. The unit cell and lamella width reported here were obtained by averaging over three images. Tunneling parameters: average tunneling current I_t = 20-40 pA, tip bias voltage V_t = -1000 mV for (**a-c**) and I_t = 20 pA, V_t = -600 mV for (**d,e**).

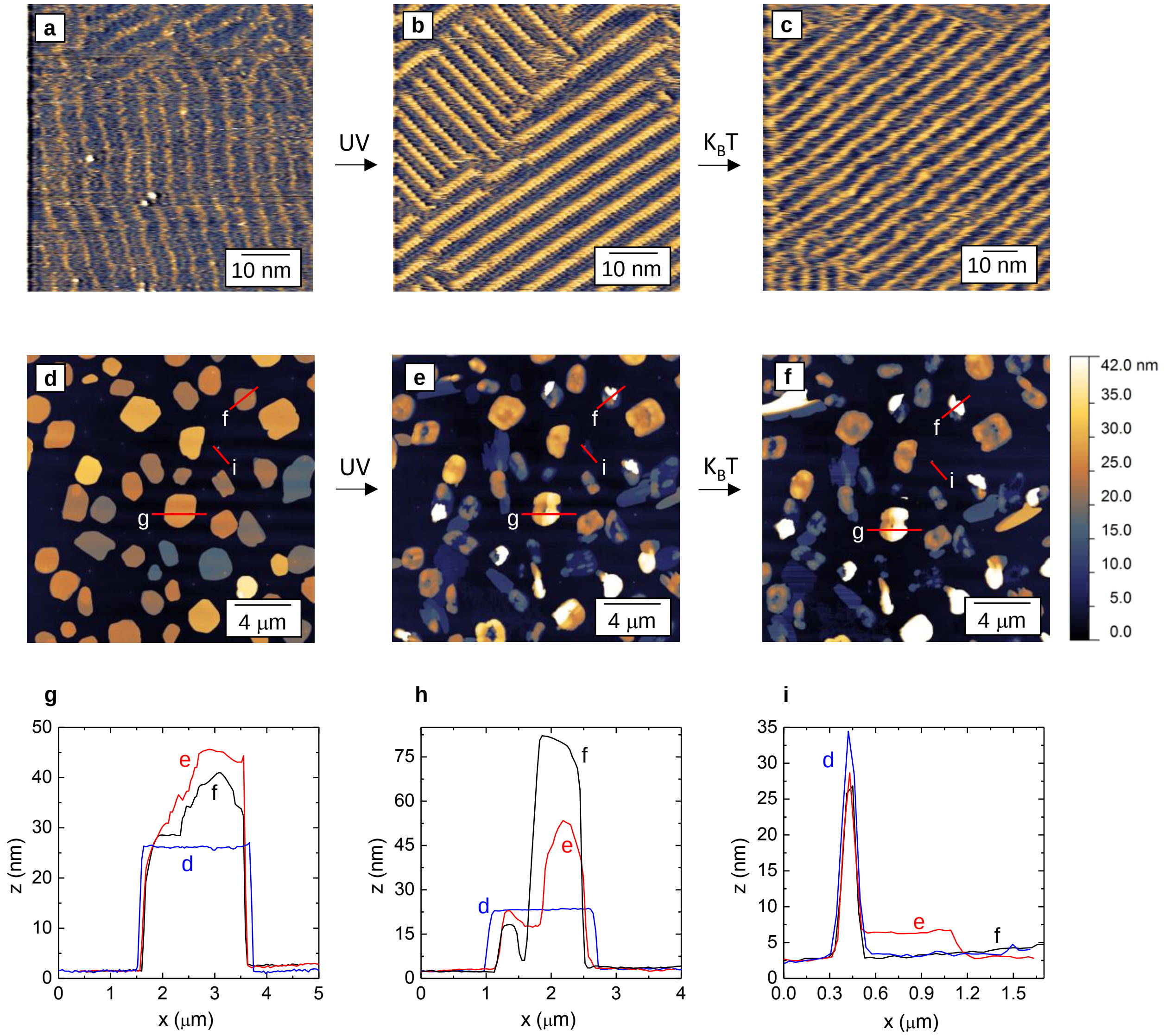

Supplementary Figure 4. Light-induced morphology reorganization of spiropyran films at different length scale. (**a-c**) Survey scanning tunnelling microscopy images of the assembly measured for a spiropyran (SP)/merocyanine (MC) film analogous to that presented in the main text, obtained by spin-coating a 0.1 mg mL^{-1} in cyclohexane solution onto HOPG. (**a**) The as-spin-coated SP film, displaying a polycrystalline lamellar structure, in which the individual domains differ on the lamellar orientation. (**b**) Image acquired on the same film after irradiation with ultraviolet (UV) light. The modified molecular ordering can be appreciated even at the ten-of-nanometer scale, by comparing (**a**) and (**b**). While in the former the edges of the lamellas are not well-defined even within a single domain, in the latter rows of precisely aligned molecules could be imaged. (**c**) The same UV-irradiated film, imaged 48 hours after irradiation, displaying

an assembly similar to the initial one. Tunneling parameters: average tunneling current (I_t) = 20 pA, tip bias voltage V_t = -1000 mV for (**a**,**c**); I_t = 20 pA, V_t = -600 mV for (b). (**d-f**) Atomic Force Microscopy study of the micro-scale evolution in the morphology of a thicker spiropyran film, as obtained by spin coating a concentrated solution (1 mg mL^{-1} in cyclohexane) onto a Si/SiO_2 (90 nm) substrate. (**d**) As-spin-coated film, revealing relatively thick structures with well-defined edges which emerge from a flat background. (**e**) Morphology of the same spot of the same film, recorded after irradiation with UV light. While the same structures could be recognized before and after irradiation, a profound rearrangement was observed in the layer morphology following the SP$\rightarrow$MC isomerization. (**f**) Morphology of the same spot of the same film, recorded 12 h after the UV irradiation with the sample kept in dark. The MC isomer is only metastable, so that it relaxes back to the SP isomer. Accordingly, the film morphology dynamically evolves even while the sample is kept in dark. The vertical scale bar is the same for images (**d-f**). (**g-i**) Height profiles measured along the red lines drawn in (**a-c**), further evidencing the dramatic changes in film morphology accompanying the isomerization. The (d,e,f) labels indicate in which image each profile was measured, being (d) before UV irradiation, (e) after UV irradiation and (f) 12 h after UV irradiation.

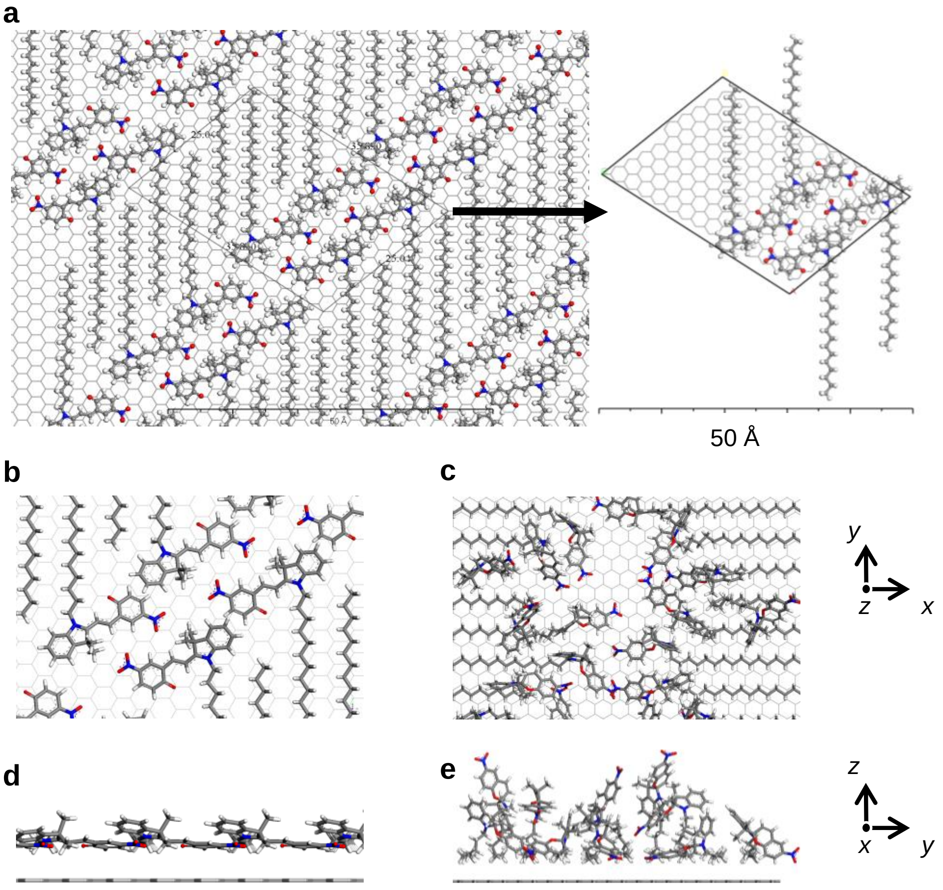

Supplementary Figure 5. Calculated assembly of spiropyran and merocyanine. (**a**) Simulated merocyanine (MC) assembly. The atomic positions have been optimized at the molecular mechanics/molecular dynamics (MM/MD) level using a re-parameterized force field. The extracted unit cell parameters are: a = 3.6 nm, b = 2.5 nm α = 107°, and A = 8.6 nm^2, corresponding to the simulation box, which is twice the experimental unit cell of the superlattice. (**b**) Arrangement of the head groups in the calculated MC assembly (top view). (**c**) Arrangement of the head groups in a representative snapshot of the time-evolving spiropyran (SP) assembly (top view). (**d**) Arrangement of the head groups in the calculated MC assembly (side view). From this image, it is clear that the positively charged part in the MC group is lifted up as compared to the graphene plane, giving rise to the vertical dipole moment and ultimately to the doping effects. Based on this calculated assembly, density functional theory calculations were performed to evaluate the charge density redistribution as well as the influence of the electrostatic potential induced by the molecular assembly on the graphene electronic band

structure, as detailed in the method section. Interestingly, it was found that the electrostatic potential associated to the charge density redistribution is a weak contribution compared to that arising from the intrinsic dipole of the monolayers. The molecule electric dipole normal to the graphene surface (calculated μ_z = 1.72 D per molecule) essentially originates from the steric hindrance of the lower methyl group, which lifts the molecule upward and breaks the symmetry of the MC isomer with respect to its molecular plane. This results in centroids of the positive and negative charges that do not coincide along z, giving rise to a relatively strong contribution to the intrinsic dipole moment of the monolayer. In addition, such leverage allows for an overlapping of neighboring MC molecules within the lamella, which maximizes the surface density of MC. Both the surface density and the (additive) electrical dipoles contribute to the strong dipole moment normal to the graphene plane**.** (**e**) Arrangement of the head groups in a representative snapshot of the time-evolving SP assembly (side view). From this image, it is clear that the SP head groups and their dipolar moments are randomly oriented.

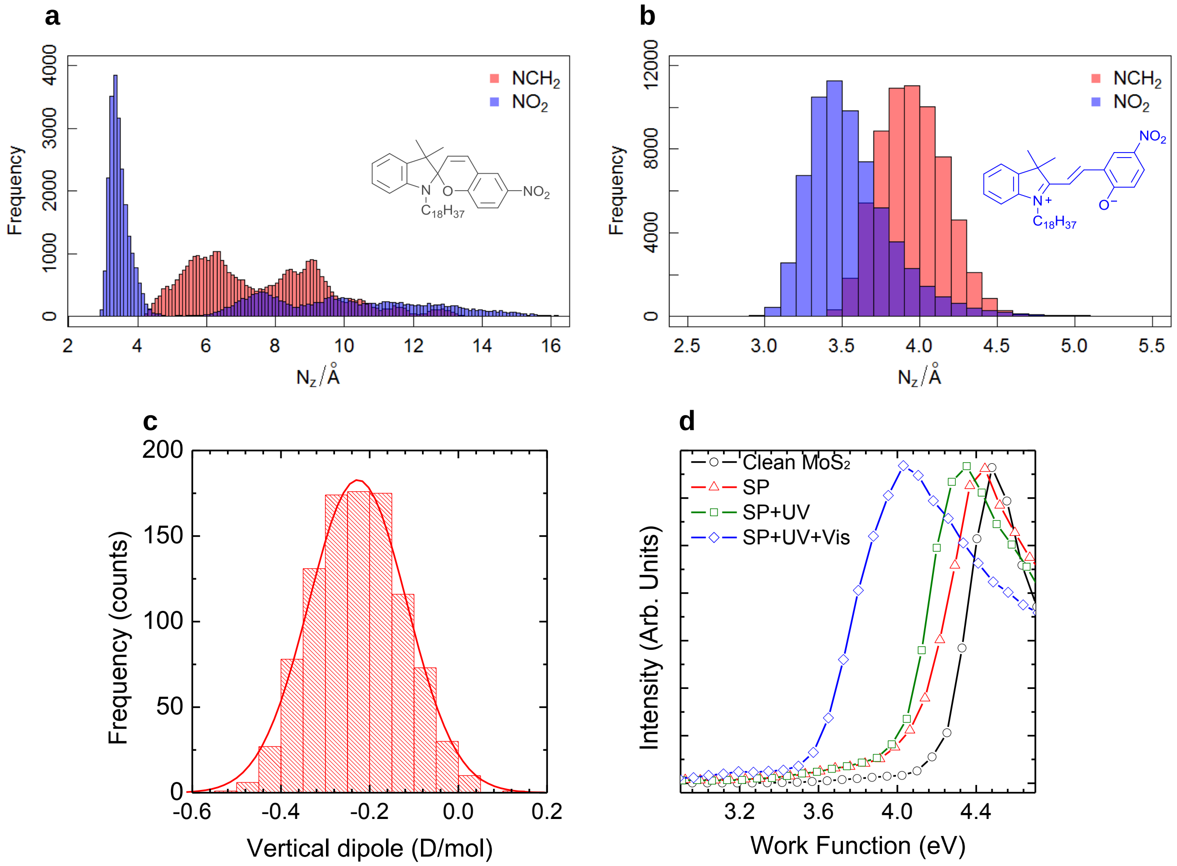

Supplementary Figure 6. Computed averaged distribution of nitrogen atoms in head groups, and corresponding electrical dipoles. The assemblies undergo thermal fluctuations and, therefore, the adsorbates explore multiple conformations at the graphene surface. In order to properly describe such time evolving situation for both the spiropyran (SP) and the merocyanine (MC) isomers, a molecular dynamics (MD) simulation of 1 ns was then performed and the coordinates of the nitrogen atoms of the molecules in the assembly were calculated at the force-field level every picosecond (1000 frames). The distributions for the so-obtained normal (z) coordinates of the nitrogen atoms are shown in (**a**) for the SP case and in (**b**) for the MC case. These distributions represent the frequency with which the different vertical positions appear in the 1000 frames, as averaged over the whole dynamic assembly at each frame, and provide useful insight on the ordering of the SP and MC head groups. (**a**) Distributions for the normal (z) coordinates of the indoline (red) and the nitro N (blue) atoms in the SP assembly. A broad seemingly bimodal distribution for the positively charged indoline N is sandwiched between the even broader distributions obtained for the N atom in the electron-withdrawing nitro group. Such

wide distribution reflects the rather large conformational flexibility of the three-dimensional head groups in the spiro isomer, and is indicative of the high level of disorder encountered in the SP case. (**b**) Distributions for the normal (z) coordinates of the indoline (red) and nitro N (blue) atoms in the MC assembly. Clearly, the positively charged indoline N is further apart from the surface as compared to the nitro group, confirming that the self-assembly lifts up the positively charged region of the MC molecule. Importantly, this MD simulations demonstrate that the lifting is robust against thermal fluctuations. As a result of this leveraging effect, a positive interfacial dipole layer forms, which is responsible for the strong *n*-type doping induced by the MC isomer. (**c**) Distribution of the vertical component normal to the graphene plane of the electric dipole of SP molecules (averaged over the entire assembly) as extracted from room temperature MD simulations in (**a**); the line is a normal distribution fit, which is centered at $\boldsymbol{\mu}_z$ = 0.23 D per molecule. (**d**) Evolution of the work function of MoS_2 as a consequence of the isomerization of the photochromic assembly. In analogy to graphite, the work function of the clean substrate (black line) is reduced by the presence of the SP assembly (red line). The SP$\rightarrow$MC isomerization induces a further, more significant reduction in the work function (blue line), which can be reverted by green light irradiation (green line). Such evolution of the work function can be fully explained on the basis of the orientation of the vertical dipoles.

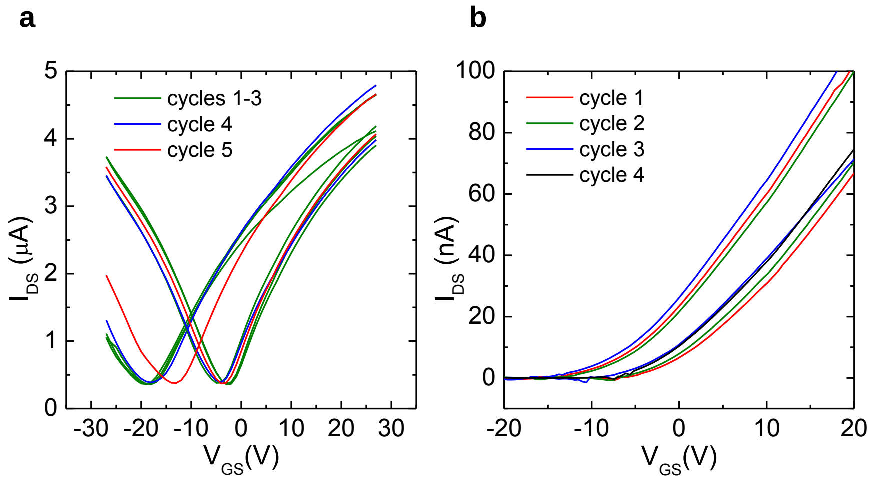

Supplementary Figure 7. Spiropyran-merocyanine cycles. (**a-b**) The stability of the spiro-mero switching against fatigue was addressed in graphene (**a**) and MoS_2 (**b**) devices, by repeating the UV/Vis light irradiation cycles described in the main text. The parameters used for the light irradiation are the same described in the Methods Section. The transfer curves were measured applying a drain source voltage V_{DS} = 10 mV for both graphene and MoS_2.

For both graphene and MoS_2, four cycles could be performed without major degradation of the switching capability. For the graphene device shown in panel (**a**), a deterioration of the effect is observed at the fifth cycle, which introduced lower *n*- type doping. It should be noted that the first three cycles were measured on the same day and were perfectly reproduced, while the fourth and fifth cycles were measured one day and one week, respectively, after the initial spin-coating, and show some fatigue.

The devices were never exposed to air: the spin-coating, the electrical measurements and the UV irradiations were performed *in-situ* in a nitrogen filled glove-box, while the green irradiation was performed *ex-situ*, but the samples were transported in a sealed chamber with nitrogen atmosphere. However, the measurement shown in panel (**a**) shows that the SP-MC film is not completely stable even in this controlled nitrogen atmosphere. We hypothesize that the deterioration measured in the devices might be caused by the protonation of the SP/MC forms.[10] This effect could be caused by the interaction of the spin-coated SP/MC films with volatile chemicals used in the nitrogen glovebox while the sample was stored.

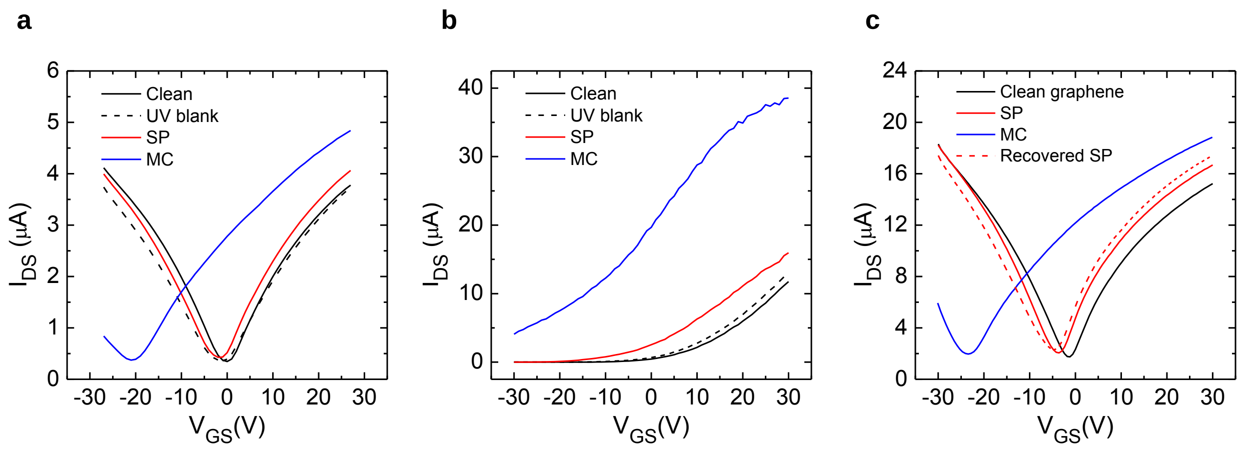

Supplementary Figure 8. Effect of UV light on clean devices and thermal recovery. In order to ensure that the reversible doping shown in the main text is not due to an artefact related to the UV irradiation itself, (**a**) clean graphene and (**b**) MoS_2 devices were UV irradiated prior to the formation of the SP layer. (**a-b**) Effect of irradiation with UV light on the electrical characteristics of clean graphene and MoS_2 devices. The electrical characteristics of the clean devices (solid black traces) were only slightly modified by the UV irradiation (dashed black traces). After this blank experiment, the effect of the SP→MC isomerization was addressed in the same devices to get a direct comparison of the magnitude of the two effects. To this goal, a SP assembly was formed on the same devices, which was found to introduce only minor changes in the electrical characteristics (solid red lines). The UV irradiation of the SP-covered flakes induced an *n*-type doping which is roughly 20 times more intense than the one observed for the clean devices (solid blue lines). The parameters of the UV irradiation (time and lamp power) were the same for clean and of SP-covered devices. (**c**) Effect of thermal recovery for a graphene device. The electrical characteristics of clean graphene are shown in black. As detailed above, the SP assembly induces a minor n-type doping (solid red curve), which is enhanced by UV-triggered SP→MC isomerization (solid blue curve). The electrical characteristics of the as-spin-coated SP assembly are recovered by leaving the device at room temperature in dark during 24 h, since the metastable SP molecules relax back to the MC isomer.

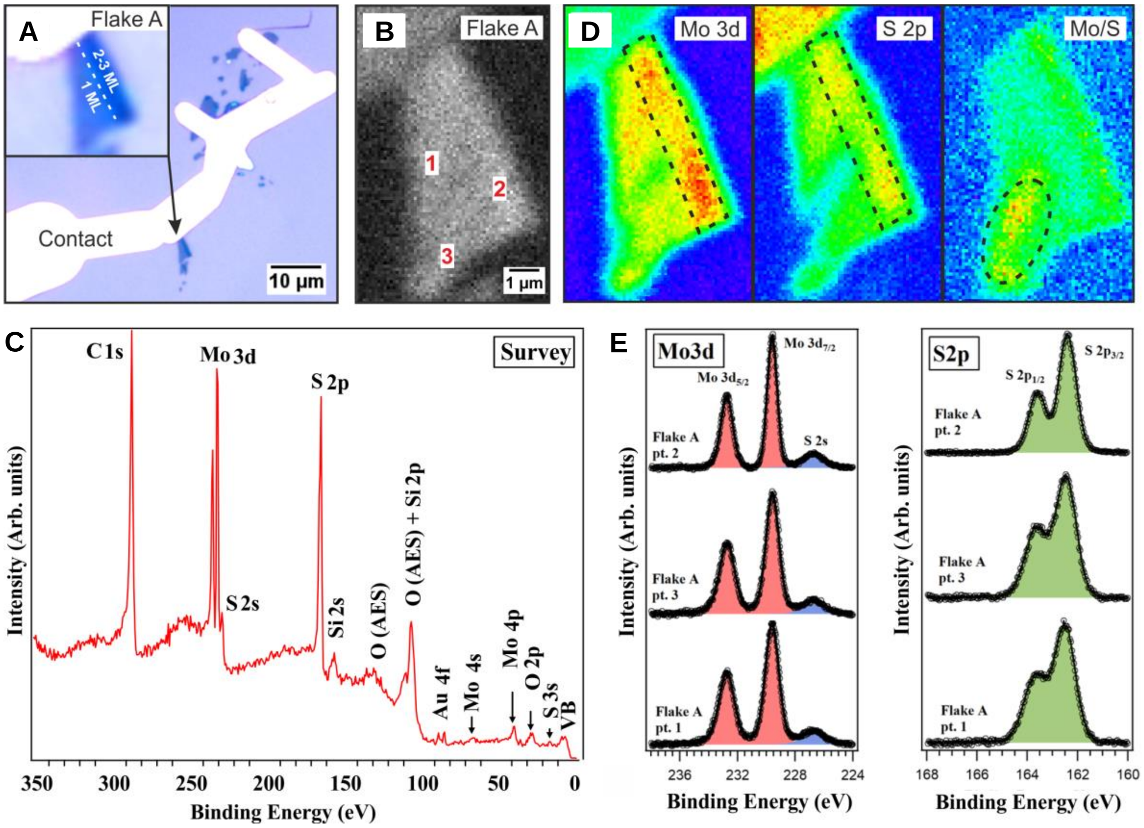

Supplementary Figure 9. XPEEM analysis of a single MoS_2 flake transferred onto a SiO_2 substrate. X-ray Photoemission Electron Microscopy (XPEEM) characterization was used to investigate the surface chemical states and relative elemental composition of the exfoliated MoS_2 flakes used for device fabrication. (**a**) Optical image of several MoS_2 flakes transferred onto a SiO_2 substrate and contacted with a gold electrode. The inset in (**a**) displays a single MoS_2 flake (denoted as flake A) that is representative for XPEEM analysis. Flake A exhibits two regions that correspond to monolayer (1 ML) and 2-3 ML thickness. (**b**) Energy-filtered XPEEM image of the flake A. The high contrast and uniformity in the XPEEM image reveals that the flake is constituted by high-quality MoS2 which makes it suitable for high-performance devices. In (**b**), three positions are indicated, where subsequent XPEEM analyses were performed. (**c**) Corresponding XPS survey micro-spectrum obtained from position 1 (see inset in (**b**)). The presence of molybdenum (Mo) and sulfur (S) in the flake is proved by the presence of the Mo 3d and S 2p peaks at binding energies (BEs) ~230 eV and ~163 eV, respectively. Besides Mo and S, contributions of silicon (Si) and oxygen (O) stemming from the underlying SiO_2 substrate are detected, underlining the atomically thin character of the MoS_2 flake. The presence of residual carbon (~1-2 ML) may be due to the exfoliation protocol and the soft lithography in order to

contact the MoS_2 flake with the gold electrode. No other residual contaminants due to the preparation protocol were detected. (**d**) Mo 3d (left panel) and S 2p (center panel) core level integrated area images, and relative elemental composition, i.e., Mo 3d : S 2p ratio (right panel). The Mo 3d and S 2p core level integrated area images exhibit a clear contrast between flake and substrate. In agreement with the optical image in (**a**), the Mo 3d and S 2p distribution intensities evidenced that the right region of the flake (marked by rectangular dotted line) is slightly thicker (i.e., 2-3 ML) with respect to the other region of the flake that is mainly constituted of a single monolayer. The difference in the thickness of the single flake is also confirmed by a small BE shift ~0. 15 eV of the Mo 3d and S 2p spectra collected in position 2 (see inset in (**b**)). From the inspection of the Mo 3d : S 2p ratio (right panel), a small contribution of surface S vacancies can be noted (marked by oval dotted line). The average stoichiometry, as calculated from the Mo 3d and S 2p spectra collected in positions 1-3, is consistent with a Mo/S ratio of 1.92, in close agreement with the expected theoretical value. (**e**) High-resolution Mo 3d and S 2p core level spectra collected in positions 1-3 after Shirley background and peak fitting. The Mo 3d spectrum consists of two peaks at 229.6 eV and 232.8 eV that correspond to Mo^{4+} 3d5/2 and Mo^{4+} 3d3/2 components of MoS_2, respectively. Neither traces of metallic Mo is found at lower BE, nor is any peak detected at around 236 eV, which could correspond to Mo6+ 3d5/2 stemming from molybdenum oxide (MoO3). In the S 2p spectrum, the typical doublet peaks of S 2p1/2 and S 2p3/2 at 163.6 and 162.5 eV, respectively, are detectable. Analogous to Mo 3d, no peaks were observed between 165 eV and 168 eV, which indicates that no oxidized S species are detectable. The absence of any oxidized species underlines once again the high-quality of the MoS_2 crystal/flake and confirms the quality of the developed exfoliation protocol and the performed soft lithography.

The XPEEM characterization was performed at the ESCA microscopy beamline of the ELETTRA synchrotron facility in Trieste (Italy). The incident soft X-ray photon beam (610 eV) was focused to a small spot with a diameter of ~150 nm at the sample surface. The sample could be scanned with an accuracy of 10 nm in front of the beam. For XPS spectra and imaging, a hemispherical 100 mm energy analyser with a 48-channel detector and an energy resolution better than 200 meV was used. XPS spectra analysis and image data were processed using IGOR Pro software. For quantitative intensity comparisons, peak areas were determined after Shirley background subtraction.

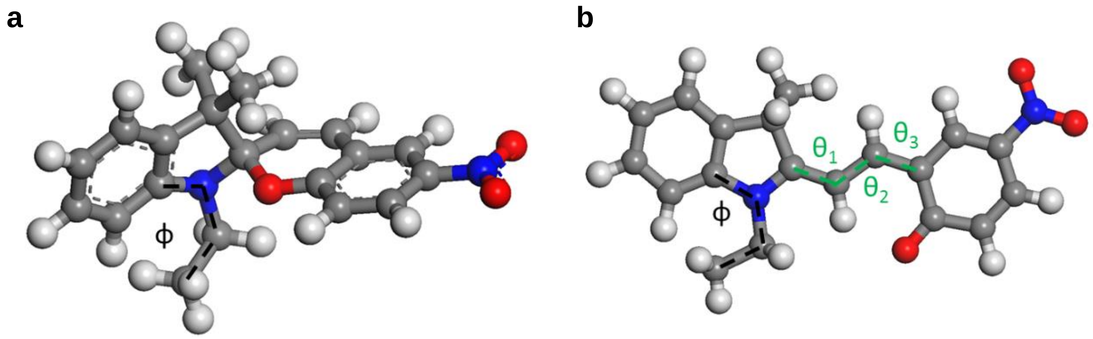

Supplementary Figure 10. Atomistic model of (**a**) spiropyran and (**b**) merocyanine moieties used for the molecular dynamics simulation.

Supplementary Note 1

Synthesis and characterization of the spiropyran derivative.

General synthetic methods, materials, and analytical techniques. Ethyl acetate, dichloromethane, petroleum ether and ethanol were distilled before usage. All other starting materials were used as received. NMR spectra were recorded on a Bruker DPX 300 Spectrometer (300 MHz for ^{1}H, 75 MHz for ^{13}C) at 25 °C using residual protonated solvent signals as internal standard (^{1}H: δ($CDCl_3$) = 7.26 ppm; δ(DMSO-d_6) = 2.50 ppm. The splitting patterns are abbreviated as follows: singlet (s), doublet (d), triplet (t), quadruplet (q), multiplet (m), and broad (br). UPLC/MS was performed with a Waters UPLC Acquity equipped with a Waters LCT Premier XE Mass Detector for UPLC-HR-MS, with Waters Alliance systems (consisting of a Waters Separations Module 2695, a Waters Diode Array Detector 996 and a Waters Mass Detector ZQ 2000). Column chromatography was carried out with silica gel (Merck 60, particle size 0.040-0.063 mm) using eluents as specified.

*Synthetic procedures. Synthesis of 1',3'-Dihydro-1'-octadecyl-3',3'-trimethyl-6-nitrospiro[2H-1-benzopyran-2,2'-(2H)-75 indole]*This compound was synthesized by adapting a literature procedure.[1] 2,3,3-trimethyl-3H-indole (2.0 mL, 12.6 mmol) was treated with 1-iodooctadecane (5.5 g, 13.8 mmol) in acetonitrile (12 mL) to give a tan suspension. While heating to 80 °C the reaction went into solution. After five days the reaction was cooled to room temperature and appeared as suspension. Acetonitrile was removed under reduced pressure and the remaining solid was washed intensively with EtOAc under sonication. This residue was dissolved in dichloromethane and washed with 1 M aqueous NaOH solution. The organic phase was dried over anhydrous $MgSO_4$ and evaporated under reduced pressure. The resulting crude product was purified by column chromatography (silica, PE/EtOAc = 96:4) to yield the product (5.1 g, 9.5 mmol, 76%) as a white solid. ^{1}H NMR (300 MHz, DMSO-d6): δ 7.97 (m, 1H), 7.85 (m, 1H), 7.62 (m, 2H), 4.44 (t, 2H), 1.81 (m, 2H), 1.53 (s, 6H), 1.22 (m, 32H), 0.84 (t, 3H); HRMS (m/z): $[M]^+$ calcd. for $C_{28}H_{49}N$, 412.3938; found, 412.3812.

*Synthesis of 1',3'-Dihydro-1'-octadecyl-3',3'-trimethyl-6-nitrospiro[2H-1-benzopyran-2,2'-(2H)-indole].*This compound was synthesized by adapting a literature procedure[2]. 1-Octadecyl-2-methylene-3,3-dimethylindoline (2.6 g, 6.3 mmol) was treated with 2-hydroxy-5-nitrobenzaldehyde (1.2 g, 6.9 mmol) in 25 mL of EtOH to give a tan suspension. While heating

the reaction to 80 °C overnight the suspension went into solution. After cooling to room temperature the mixture was diluted with water and EtOAc and separated. The aqueous phase was back extracted with EtOAc. Combined the organic layers and washed with water and saturated aqueous NaCl solution. The organic phase was dried over anhydrous $MgSO_4$ and evaporated under reduced pressure. The resulting crude product was purified by column chromatography (silica, PE/EtOAc 9:1) to yield the product (1.73 g, 3.1 mmol, 50%) as a white solid. Characterization data agree with the literature[2]. ^{1}H NMR (300 MHz, $CDCl_3$): δ 8.03 (d, J = 2.7 Hz, 1H), 8.00 (m, 1H), 7.19 (dt, J = 7.5 Hz, 1.2 Hz, 1H), 7.09 (dd, J = 7.2 Hz, 1.2 Hz, 1H), 6.90 (d, J = 10.2 Hz, 1H), 6.87 (dt, J = 7.2 Hz, 0.9 Hz, 1H), 6.74 (d, J = 8.7 Hz, 1H), 6.57 (d, J = 7.5 Hz, 1H), 5.86 (d, J = 10.5 Hz, 1H), 3.14 (m, 2H), 1.26 (m, 32H), 1.19 (s, 3H), 0.89 (t, J = 6.9 Hz, 3H); HRMS (m/z): $[M]^+$ calcd. for $C_{36}H_{52}N_2O_3$, 561.4051; found, 561.4059.

Supplementary Note 2

Molecular mechanics/dynamics simulations and density functional theory calculations.

To assess the role of a spiropyran/merocyanine (SP/MC) molecular adlayer on the WF of graphene, we have deployed a three-step procedure including: i) parameterization of a classical force field against quantum-chemical calculations for the two isomers; ii) molecular mechanics/dynamics (MM/MD) simulations of their supramolecular 2D assembly on graphene; and iii) electronic structure calculations of the hybrid graphene-adlayer systems at the Density Functional Theory (DFT) level. Strictly step iii) of the procedure described above was only performed for the MC case, since a well-ordered assembly was not encountered. In the SP case, the effect on the electronic properties of graphene was evaluated through the study of the time evolving vertical dipoles extracted by the MD simulations.

Force field parameterization. An all-atom description was used for the individual molecules, i.e., all hydrogen atoms were taken explicitly into account. The calculations were performed using a modified version of the Dreiding Molecular Mechanics force-field, which was selected because the additive contributions to the total energy can easily be selectively tuned to reproduce either experimental data or the results from higher-level theory calculations. Namely, it is important to treat quantitatively both bonded degrees of freedom, such as torsion angles that control molecular conformation, and non-bonded degrees of freedom, including electrostatics, which

govern intermolecular interactions. Specifically, the original Dreiding force field was re-paramaterized to reproduce the torsional potential *φ* around the N-C(H_2) bond of the SP compound against reference MP2/cc-pvdz results (Supplementary Fig. 10). In practice, this is done by adjusting the energy constants in the torsional energy term (sum of cosines) so as the energy minima/maxima and energy barriers are properly reproduced, i.e. angle deviations below 15° at the extrema and barrier height within less than 2 kcal mol^{-1}. In the case of the MC compound, we focused our attention on the torsional potential φ around the N-C(H_2) as well as the dihedral angles θ_1, θ_2, andθ_3, which control the planarity of the MC moiety. For the aliphatic chains of the molecules, the COMPASS force field (FF) was used for all the atomic charges, except for the first –CH_2– unit close to the SP/MC functional group. While the COMPAS force field has been demonstrated to provide an accurate prediction of structural and thermodynamic properties for alkanes and related organic materials[3], it is less appropriate for the aromatic SP/MC heads.

For these moieties, we instead relied on atomic charges calculated at a higher quantum-chemical level of theory, specifically using electrostatic potential (ESP) charges[4] obtained on the basis of MP2/cc-pvdz optimized geometries. [5] Then, chemically equivalent atoms were set with equal (averaged) charges. All ab initio calculations were performed using the Gaussian09 package while the MM/MD calculations were performed with the Materials Studio 6.0 package using our fine-tuned Dreiding force-field.

A.2 Evaluation of the WF shift at DFT level We have performed periodic boundary conditions (PBC) Density Functional Theory calculations using the SIESTA software package [6] to evaluate the charge density redistribution as well as the influence of the electrostatic potential induced by the molecular assembly on the graphene electronic band structure. The PBE functional has been chosen for the description of exchange and correlation (GGA). The work function of a substrate Φ is defined as the energy required to extract an electron from the bulk to the vacuum level:

$$\Phi = V_\infty - E_\mathrm{F} \tag{1}$$

where V_∞ is the electrostatic potential in vacuum and E_F is the Fermi level of the bulk (here graphene). The contribution of the interface potential to the work function shift can be estimated via the charge density difference at the interface:

$$\Delta\rho(\mathrm{z}) = \rho_\mathrm{sys} - (\rho_\mathrm{assembly} + \rho_\mathrm{slg}) \tag{2}$$

where ρ_{sys}, $\rho_{assembly}$, and ρ_{slg} are the charge density of the whole system (interface), the molecular assembly and the graphene layer, respectively. The electrostatic potential ΔV_E associated to the charge density difference at the interface $\Delta\rho$ is obtained by a numerical integration of the Poisson equation:

$$\frac{d^2V_\mathrm{E}}{dz^2} = -\frac{\Delta\rho}{\varepsilon_0} \tag{3}$$

The work function shift can be expressed as a combination of two contributions:

$$\Delta\Phi = \Delta V_\mathrm{assembly} + B.D. = \Delta V_\mathrm{assembly} + \Delta V_\mathrm{E} + \Delta V_\mathrm{slg} \tag{4}$$

where $\Delta V_{assembly}$ is the shift of the electrostatic potential induced by the intrinsic dipole moment of the molecular assembly, and *B.D.* is the potential change at the interface due to the adsorption of the molecular assembly [7]. The latter can be decomposed in two terms, namely the geometric rearrangement of the substrate and the electronic reorganization resulting from the adsorption of the molecular assembly on graphene. Upon physisorption of the molecular assembly on graphene, it is expected that the graphene geometry is almost unperturbed; i.e., no geometric restructuration of the carbon atoms occurs ($\Delta V_\mathrm{slg} = 0$). Therefore, the work function shift, $\Delta\Phi$, can be expressed in terms of the local electrostatic potential associated to the charge density redistribution ΔV_E between graphene and the monolayer, and the contribution of the molecular assembly $\Delta V_\mathrm{assembly}$. By computing the potential profile across the molecules in the direction normal to the graphene plane (*z*-axis), $\Delta V_\mathrm{assembly}$ can also be calculated from the electric dipole of the molecules. Indeed, in the Helmholtz model, the assembly contribution is directly proportional to the electric dipole of the molecules along the axis normal to the graphene surface, μ:

$$\Delta V_\mathrm{assembly} = \frac{\mu \mathrm{e}}{\varepsilon_0 S} \tag{5}$$

where *e* is the elementary charge, ε_0 the vacuum permittivity, and *S* the surface area of the unit cell. Note that by using the effective dipole of the molecule from calculations performed on the assembly (using periodic boundary conditions), depolarization and image charge effects are automatically built in.

MC assembly. Molecular dynamics simulations. Prior to the construction of the molecular assemblies, MD calculations were performed in order to determine the geometrical parameters of the MC monolayer adsorbed on the graphene surface, the relative stability of different orientations of the molecules with respect to the graphene layer, and between molecules (the

relative orientations and shifts of the functional groups and alkyl chains, the inter-digitation of the alkyl chains, etc). Assemblies of the MC derivatives were built atop the graphene surface, which consists in a frozen layer of 12160 carbon atoms (~13.7 × 23.4 ≈ 320.6 nm²) (as the geometry is expected to be unperturbed upon physisorption of the molecules). As a result, the starting 2D adlayer has four stacks of 16 (i.e., 64 altogether) MC molecules initially placed at ~0.3 nm from the graphene surface (alkyl chains parallel to the zigzag main axis) with adjacent MC derivatives in antiparallel configuration; the alkyl chains are interdigitated with a distance of 0.43 nm.

A full energy minimization of the assembly-SLG systems was performed in vacuum, using periodic boundary conditions (PBC) and the Conjugate-Gradient algorithm, until the RMS force was less than 5×10^{-3} $kcal^{-1}$ mol/Å with energy convergence of 1×10^{-4} kcal mol^{-1} between steps. Series of 25-ps-quenched dynamics (NVT, T = 50, 100, …, 300 K, quench frequency = 1 ps) were then launched at different temperatures until the energy between quenched dynamics no longer decreases.

*MC assembly. Evaluation of the WF shift at the DFT level***.** In order to considerably reduce the computational time, the linear alkyl chains have been removed in the subsequent DFT electronic structure calculations, i.e., $C_{18}H_{37}$ have been replaced by CH_3 groups. Indeed, the saturated chains should not contribute to the doping of graphene. Thus, the unit cells consist in a graphene layer of 326 carbon atoms and 4 MC moieties on top of it. No further optimization of the unit cell has been performed at the DFT level. The different contributions in Eq. (4) have been calculated for the MC derivative following the methodology described above. With the aid of DFT calculations, we could estimate that the MC assembly would induce a shift of ΔWF= -0.4 eV.

SP Assembly. Molecular dynamics simulations. The SP derivative has a stereocentric carbon atom and is, thus, a chiral molecule (Supplementary Fig. 10). As a result, the molecule adopts a 3D conformation on the graphene surface, in contrast to the MC derivative that is 2D (planar molecule). Hence, spontaneous self-assembly of the SP molecules is likely exclusively driven by the alkyl chains. The physisorption of a single SP molecule on graphene surface has first been simulated with different starting conformations (rotation of the Φ angle, see Supplementary Fig. 10) and energy minimization plus quenched dynamics have been performed in order to identify the lowest energy conformations of the SP molecule on the graphene surface. The simulations

have been performed on both enantiomers (R and S) using the fine-tuned DREIDING force-field. The formation of the SP monolayers has next been investigated, adopting the same approach used for the MC derivative. Namely, the lowest-energy conformations have been used as building blocks to build the assemblies with different organizations, including mixture of low-energy conformations, interdigitation of alkyl chains, racemic or chiral assemblies, etc. Unfortunately, the quenched dynamics simulations failed in providing well-organized 2D architectures, likely because of the multiple possible arrangements of the aromatic heads of the chiral molecules. We thus turned to a different approach, whereby SP monolayers were prepared using input from STM measurements. From the experimental data, alkyl chains are physisorbed, forming 2D crystalline structure on the graphene surface (Fig. 1). The conjugated core of the SP moiety leads to bright spots, which are aligned perpendicular to the alkyl chain axes (dark regions). The SP isomers can apparently organize in contiguous lamellae adopting either head-to-head or head-to-tail (Supplementary Fig. 3) configurations.

Usually, racemic mixture of a chiral compound dropped on HOPG leads to the formation of a monolayer with various domains (chiral or racemic)[8,9]. Here, no chiral domains can be distinguished on the STM images. Therefore, we assume the monolayers are formed with a statistical ratio of both enantiomers. Considering the geometrical features extracted by the STM images (Fig. 1e), a non-interdigitated SP monolayer has been simulated. We started with a geometry optimization of a SP dimer (with both enantiomer in head-to-head fashion) and use the relaxed structure as a building block for the preparation of the monolayer. In order to preserve that organization, we then ran MD simulations where the tails of the alkyl chains (ten CH_2 units) are spatially constrained. Note that we used in these calculations the upper estimate within the experimental error bar for the value of parameter *a* (6.1 nm), so as to leave more space to the head groups. This approach thus enforces a self-organization driven by the alkyl side chains in line with the STM data, while allowing the aromatic cores to fully explore their (complex) configurational space.

MD simulation runs over 1 ns yield for SP broad distributions of the aromatic head group orientation that translate into small (compared to MC) interfacial dipoles, with average and standard deviations of 0.23 D and 0.11 D, respectively (see Supplementary Fig. 6).

Supplementary References

1. Kim, S.-H. *et al.* Crystalline-state photochromism and thermochromism of new spiroxazine. *Dyes Pigm.* **53,** 251–256 (2002).
2. Sasaki, H., Kobayashi, S., Itoh, Y. & Osa, T. Photoinduced Resistance Change across Poly(vinyl chloride)/Spirobenzopyran Membrane. *Chem. Lett.* **19,** 555–558 (1990).
3. Sun, H. COMPASS: An ab Initio Force-Field Optimized for Condensed-Phase Applications Overview with Details on Alkane and Benzene Compounds. *J. Phys. Chem. B* **102,** 7338–7364 (1998).
4. Besler B. H., Merz K. M., J. & A., K. P. Atomic charges derived from semiempirical methods. *J. Comput. Chem.* **11,** 431–439 (1990).
5. Frisch, M. J. *et al.* Gaussian 09, Revision D.01. Gaussian, Inc., Wallingford, CT, 2009.
6. Soler, J. M. *et al.* The SIESTA method for ab initio order-N materials simulation. *J. Phys. Condens. Matter* **14,** 2745 (2002).
7. Heimel, G., Romaner, L., Bredas, J. L. & Zojer, E. Organic/metal interfaces in self-assembled monolayers of conjugated thiols: A first-principles benchmark study. *Surf. Sci.* **600,** 4548–4562 (2006).
8. Minoia, A. *et al.* Assessing the Role of Chirality in the Formation of Rosette-Like Supramolecular Assemblies on Surfaces. *Chem. Commun. (Camb).* **47,** 10924–10926. (2011).
9. Minoia, A. *et al.* Design of Efficient Sergeant Molecules for Chiral Induction in Nano-porous Supramolecular Assemblies. *RSC Adv.* **5,** 6642–6646 (2015).
10. Klajn, R. Spiropyran-based dynamic materials. *Chem. Soc. Rev.* **43,** 148–184 (2014).
